# Intraspecific support for the climate variability hypothesis: oxidative damage in lizards after acute temperature exposure

**DOI:** 10.1242/jeb.251040

**Published:** 2025-09-26

**Authors:** Benjamin D. Haussmann, Nicole A. Joseph, Tiffany R. Hegdahl, Kayla E. Lichtner, Redeit N. Woldebirhan, Benjamin G. Travis, Garret P. Peterson, Travis R. Robbins, Mark F. Haussmann

**Affiliations:** ^1^ Department of Biological Sciences, Auburn University, Auburn, AL 36849, USA; ^2^ Department of Biology, University of Nebraska Omaha, Omaha, NE 68182, USA; ^3^ Department of Biology, Bucknell University, Lewisburg, PA 17837, USA; ^4^ Department of Animal Science, University of Nebraska Lincoln, Lincoln, NE 68583-0908, USA

**Keywords:** Ectotherm, Temperature, 8-OHdG, Oxidative stress, Climate variability hypothesis, Mitochondria

## Abstract

Ectotherms face mounting challenges from climate variability. The climate variability hypothesis predicts that species from more variable environments will exhibit greater physiological resilience, but this has been largely untested within a species. Because ectotherm metabolic rates increase with temperature, mitochondrial function and its byproduct, reactive oxygen species, may play key roles in this thermal resilience. This study examined how temperature affects oxidative damage in prairie lizards (*Sceloporus consobrinus*) from three populations (northern, central and southern) along a latitudinal gradient in two separate experiments on different individuals. In the first experiment, lizards were exposed to day-long thermal exposures (18°C and 37°C). Oxidative damage, measured as 8-hydroxy-2'-deoxyguanosine (8-OHdG), increased in only the central and southern populations after cold exposure. Notably, the northern population, consistent with predictions of the climate variability hypothesis, showed no increase, suggesting possible adaptations to mitigate cold-induced oxidative damage. In the second experiment, we tested whether oxidative damage was triggered by cold exposure or subsequent rewarming. Again, northern lizards showed no change, whereas southern lizards increased damage with faster rewarming rates. Finally, we found that 8-OHdG decreased 24-h after the cold exposure and rewarming, indicating it may be reversible. Collectively, these results provide the first intraspecific evidence for the climate variability hypothesis in a vertebrate ectotherm. This suggests that cold-adapted lizards possess mechanisms to buffer oxidative damage, emphasizing the role of mitochondrial function and oxidative resilience in shaping thermal tolerances.

## Teaser

**Summary:** Southern and northern populations of prairie lizards show different responses of oxidative damage levels following different thermal exposures.

## INTRODUCTION

Species with large geographic ranges encounter diverse environmental conditions, including significant temperature variability (Ferguson and Brockman, 1980; Sokolova, 2023). The ability of species to persist across such gradients depends, in part, on their physiological capacity to cope with varying thermal environments. The biological impacts of environmental temperature are particularly profound in ectotherms, which make up the vast majority of animal biodiversity (Wilson, 2010). Because they rely on external heat sources for thermoregulation, their body temperature fluctuates directly with environmental temperature (Sokolova, 2023). The climate variability hypothesis suggests that species inhabiting more variable climates, typically at higher altitudes and latitudes, evolve broader physiological thermal ranges, whereas those in more thermally stable environments develop narrower tolerances (Addo-Bediako et al., 2000; Janzen, 1967; Polato et al., 2018). In support of this idea, comparative studies show that species at higher latitudes tend to exhibit wider thermal breadths than those from more stable, tropical regions (Dewenter et al., 2024). Thus, temperature can act as a powerful selective force, driving physiological differentiation and even speciation (Seebacher et al., 2015).

Traditionally, the climate variability hypothesis has focused on thermal tolerance breadths, the range of temperatures at which an organism can survive, across wide latitudinal ranges (Janzen, 1967). More recently, the climate variability hypothesis has been extended to thermal tolerance performance, or how physiological traits perform over a range of temperatures (Ghalambor et al., 2006). This shift reflects the importance of understanding the profound impact of temperature across all biological levels, moving beyond organismal survival to focus on the molecular and physiological processes underlying variation in survival. For example, temperature can impact survival indirectly through its effect on biochemical reactions, membrane stability, signal transduction and even DNA integrity (Somero et al., 2016). In response to this, recent studies on the climate variability hypothesis have explored physiological performance traits including such as rates of dehydration (Bovo et al., 2023), seed production (Widick et al., 2025), photosynthesis (Perez et al., 2023), mitochondrial function (Havird et al., 2020) and metabolic rate (Pacioni et al., 2023).

Of particular interest are the effects of temperature on metabolism, a complex, multivariable process with numerous cellular pathways and byproducts (Pettersen et al., 2018). At the heart of metabolism are mitochondria, which play a crucial role in ATP synthesis and metabolic regulation. In ectotherms, maintaining mitochondrial integrity and metabolic homeostasis under thermal variation is essential for organismal performance and fitness (Sokolova, 2018, 2023). Beyond their role in energy production, mitochondria function as cellular stress sensors and signaling hubs, transmitting stress signals within and between cells (Picard and Shirihai, 2022). One important signaling mechanism involves reactive oxygen species (ROS). ROS are produced during electron flow through the mitochondrial electron transfer system when some electrons escape and prematurely react with oxygen. Despite their important physiological signaling functions at low levels (Dröge, 2002), excessive ROS can cause oxidative damage to DNA, proteins and lipid membranes, impairing cellular function (Monaghan et al., 2009). The accumulation of oxidative damage can have profound consequences for growth, reproduction and aging, ultimately shaping life history strategies (Meniri et al., 2022; Metcalfe and Alonso-Alvarez, 2010; Monaghan et al., 2009). As a result, selection may act at multiple levels of the metabolic and oxidative phosphorylation process – from the production of ROS to the limitation of their spread, and finally, to the repair and removal of damaged structures.

Mitochondria and, in turn, ROS production and oxidative damage, are all affected by temperature. For example, temperature can affect mitochondria both directly by altering membrane fluidity and enzyme activity and indirectly by increasing cellular ATP demand (Somero et al., 2016). In addition, a growing body of research in vertebrate and invertebrate ectotherms suggests that exposure to temperatures outside a species' optimal range, whether excessively hot or cold, leads to increased oxidative stress and cellular damage (reviewed by Ritchie and Friesen, 2022). Furthermore, a rapid increase in body temperature following cold exposure, a thermal rewarming, has also been linked to increased ROS production and oxidative damage (Madeira et al., 2016). Although a recent study has explored mitochondrial function in the climate variability hypothesis framework (Havird et al., 2020), work on latitudinal variation in temperature-induced oxidative damage is lacking.

To date, the climate variability hypothesis has largely focused on interspecific comparisons. Extending the hypothesis further to examining how populations spanning latitudinal gradients differ in their physiological responses to thermal exposures would provide novel insight into thermal adaptation and evolutionary responses to climate variability in vertebrate ectotherms. Despite growing recognition that within-species variation in thermal physiology can significantly influence population-level vulnerability to climate change (Bennett et al., 2019), many studies still assume species-level thermal niches without testing for population-specific adaptations. Temperature imposes strong selective pressures that shape phenotypic responses along geographic clines (Lardies et al., 2010), leading to intraspecific variation in physiological traits across populations occupying different thermal environments (Gaitán-Espitia and Nespolo, 2014). Investigating how populations of a widely distributed species adapt to local climatic conditions provides key insights into the mechanisms underlying thermal tolerance and evolutionary adaptation in response to environmental change (Seebacher et al., 2015).

The *Sceloporus* lizards offer an ideal study system to study the climate variability hypothesis within a species as they span a wide latitudinal range and experience varying environmental conditions. In this study, we used three populations (northern, central and southern) of prairie lizards (*Sceloporus consobrinus*)*,* which have distinct thermal environments. The northern population experiences a colder average annual temperature and the highest inter-daily temperature fluctuations across the year, with the southern population having the smallest and the central showing intermediate inter-daily variability (Table 1). In addition, previous work has found population differences in body condition, growth rate, clutch mass and metabolic rates (Haussmann et al., 2025; Robbins and Hegdahl, 2024; Table 1). Taken together, the distinct thermal environments and intraspecific variation among these populations offer an ideal system to test the climate variability hypothesis within a species.

**Table 1.** Population information and population lizard characteristics for three populations of the prairie lizard, *Sceloporus consobrinu*s, in the USA

Population	Population information		Lizard characteristics			
	Latitude, Longitude	Annual average temperature (°C)	Body condition (g mm^−1^)	Weekly growth rate (mm)	Clutch mass (g)	Fed metabolic rate (ml CO_2_ h^1^)
Northern	36°22′48′′N, 94°58′58′′W	16.0±8.6	8.4±0.24^a^	0.11±0.01^a^	3.2±0.42^a^	1.28±0.08^a^
Central	34°07′54′′N, 94°40′47′′W	16.7±8.0	7.7±0.17^ab^	0.09±0.20^a,b^	2.1±0.21^b^	1.03±0.07^b^
Southern	31°54′26′′N, 95°54′07′′W	19.1±7.3	7.5±0.24^b^	0.05±0.01^b^	1.7±0.16^b^	0.99±0.08^b^

The northern population exhibits the highest day-to-day seasonal variability in terms of temperature, whereas the southern population exhibits the lowest variation (Levene's test, *P*<0.001). Data are from the National Centers for Environmental Information (NCEI) database. For body condition, weekly growth rate and clutch mass (means±s.e.m.; Robbins and Hegdahl, 2024) and fed metabolic rate (estimated marginal means±s.e.m.; Haussmann et al., 2025), different letters denote statistically significant differences among populations.

Here, we investigated temperature-induced oxidative damage using a common garden design. This allowed us to examine plastic responses to acute thermal change and evolutionary differences among populations of the wide-ranging prairie lizard, *Sceloporus consobrinus*. Our experimental aims were as follows. First, we investigated whether populations differ in oxidative damage in response to cold and warm thermal exposures. We predicted both heat and cold exposures to induce oxidative damage. However, based on the climate variability hypothesis, we predicted that the northernmost populations, experiencing greater thermal variability, would exhibit less oxidative damage accumulation after thermal exposures than their southern counterparts (Addo-Bediako et al., 2000). Second, we aimed to distinguish whether oxidative damage following cold exposure is caused by the cold temperature itself or the subsequent thermal rewarming. Both possibilities are supported in the literature, but studies are rarely designed to distinguish between them or assess whether both processes contribute to damage independently (but see Salin et al., 2015). Finally, we examined whether temperature-induced oxidative damage persists or declines after a cold exposure and subsequent rewarming. To our knowledge, no studies have conducted a longitudinal assessment to track the recovery of oxidative damage following thermal exposure.

## MATERIALS AND METHODS

### Animal care and collection

**Fig. 1.**
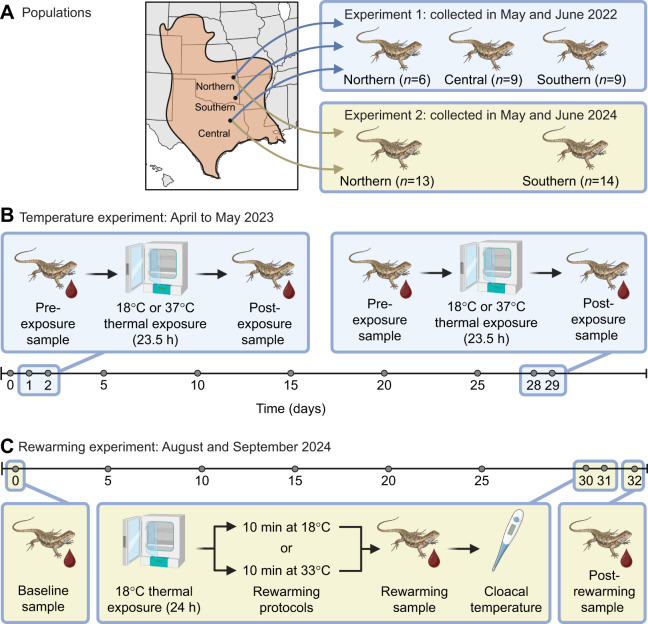
**Schematic diagram of the experimental design.** (A) Lizards were collected from their species distribution (orange area) across a latitudinal gradient in the USA (Table 1) in 2022 for the temperature experiment and 2024 for the rewarming experiment and brought into the lab. (B) The temperature experiment was performed on lizards from northern, central and southern populations. All lizards experienced two 23.5 h thermal exposures (18°C or 37°C) that were 27 days apart using a cross-over design. Blood samples were taken directly before (pre-exposure) and after (post-exposure) the thermal exposure. (C) The rewarming experiment was performed on a different set of lizards from the northern and southern populations. A baseline sample was taken 30 days before a 24 h, 18°C thermal exposure. After the thermal exposure, lizards were subjected to one of two rewarming protocols: a fast rewarming protocol (moved to a 33°C incubator) or a slow rewarming protocol (moved to an 18°C incubator). After 10 min, blood samples were taken from lizards from both groups and then body temperatures were recorded. Because larger-bodied lizards warm more slowly than smaller individuals, and because blood draw time varied slightly between lizards, the combination of the rewarming protocol, the amount of time spent rewarming and body mass created the desired substantial variation in rewarming rates. A post-rewarming sample was taken 24 h later to examine longitudinal changes in oxidative damage. Created in BioRender by Haussmann, M., 2025, https://BioRender.com/u98t7ak. This figure was sublicensed under CC-BY 4.0 terms.

Adult prairie lizards, *Sceloporus consobrinus* Baird & Girard 1854, were collected from three sites across a latitudinal gradient, each with distinct thermal environments (Table 1, Fig. 1A). For simplicity, we will refer to the three populations as northern, central and southern. Owing to facility space restrictions and concerns regarding statistical power, only the females from each population were used. Although our populations only span 4.4° latitude, there are still significant differences in average annual temperature. More importantly, the standard deviation of daily mean temperatures across the year is also significantly different among populations, with the northern population being the most variable (Table 1). To provide more information about how lizards in these populations differ, Table 1 includes data from previous studies on these same populations, which have found differences in morphology and physiology from common garden experiments. Lizards were collected in May and June of both 2022 and 2024 and were brought back to the University of Nebraska Omaha Animal Care Facility, where they were housed in terraria (60×42×34 cm) with paper towel substrate. Heat lamps were active from 09:00 to 15:00 h to allow lizards to thermoregulate (substrate temperature range: 24–34°C) at a room temperature of 24°C on a 12 h:12 h light:dark cycle. Previous work on the same populations of lizards, after laboratory acclimation, found no differences in preferred body temperature, suggesting that lizards are not acclimating differently to the common garden conditions (Robbins and Hegdahl, 2024). Each lizard was provided five crickets, supplemented with vitamin and calcium powder (Repashy Ventures Inc.), three times a week and water was provided *ad libitum*. All experiments and procedures were approved by the University of Nebraska Omaha Institutional Animal Care and Use Committee (IACUC; protocol 22-001-01) and animal collection was authorized by the respective state permits (OK and TX). The University of Nebraska Omaha Animal Care and Use Program is AAALAC International accredited. All procedures were in compliance with recommended ARRIVE guidelines (Percie Du Sert et al., 2020). We have not used AI-assisted technologies in creating this article.

Note that the first and second experiments, conducted in 2022 and 2024, respectively, represent distinct replications using different lizard cohorts and slight methodological modifications. These differing conditions across experiments (e.g. variations in pre-experiment captivity duration and length of thermal exposure) therefore allowed for an assessment of the robustness and generalizability of our findings. In the first experiment, lizards from all three populations (northern, central and southern) were used. Based on the results from this experiment, which showed no significant differences between the central and southern population (see Results), the second experiment was designed to focus solely on northern and southern populations to bolster sample size for this comparison. Further details on each experiment are provided below.

### Temperature experiment: effect of warm and cold thermal exposures on oxidative damage

The temperature experiment was conducted in April and May 2023 (Fig. 1B). Blood cell oxidative damage to DNA was measured for lizards from all three populations in response to two distinct thermal exposures. The two experimental temperatures were based on preferred body temperatures and critical thermal limits for *Sceloporus* lizards. Specifically, experimental temperatures were chosen outside of the preferred temperature range of *S. consobrinus* (28.6°C to 35.9°C; Robbins and Hegdahl, 2024), while avoiding extreme experimental temperatures that approached the critical thermal limits for other species of *Sceloporus* lizards (minimum: 8.6±5.5°C, maximum: 42.4±1.8°C; Plasman et al., 2024). We chose 18°C for the low temperature because it was located in the middle of the lower thermal window (8.6°C to 28.6°C) and 37°C for the high temperature because it was at the low end of the upper thermal window (35.9°C to 42.4°C). Although lizards can endure lower temperatures by entering states of reduced metabolic activity, upper critical temperatures are more lethal because high temperatures can cause irreversible damage and mortality (Cowles and Bogert, 1944).

Lizards were exposed to two distinct day-long thermal exposures (18°C and 37°C), conducted 4 weeks apart to ensure full recovery. We employed a standard crossover design, where each individual experienced both temperatures, but in different sequences to control for potential order effects. Specifically, half of the lizards were randomly assigned to experience the 18°C temperature during the first exposure (days 1–2), followed by the 37°C during the second exposure (days 28–29); the other half experienced 37°C first, followed by 18°C (Fig. 1B). Although we acknowledge that lizards would not naturally experience a constant temperature for an entire day, body temperatures of 18°C and 37°C in a single 24-h period are not uncommon for these populations. Our aim was to determine the response of oxidative damage to a simple, constant experimental thermal treatment. Although short in duration, previous work in ectotherms reported that a 24-h temperature exposure allows for significant changes in mitochondrial gene expression (Jin et al., 2022).

On the morning (09:00 h) of a thermal treatment, lizards were fed a cricket weighing between 3% and 5% of the lizard's body mass. This ensured all lizards experienced the temperature exposure in a fed state, as previous work in an ectotherm demonstrated that fed versus fasted states can affect oxidative damage (Butler et al., 2016). Only lizards that consumed the cricket and were in a digestive state were included in the study. At 13:00 h, pre-exposure blood samples were collected (mean±s.d. sampling time 3.7±1.6 min; see Materials and Methods, Blood sampling and processing). Afterwards, lizards were placed in a 200 ml respirometry chambers housed within an incubator set to one of the two exposure temperatures, where they remained for 23.5 h (Fig. 1B). The post-exposure blood sample was taken as quickly as possible after the lizard was removed from the temperature-controlled chamber (sampling time 3.6±1.7 min).

### Rewarming experiment: effect of cold exposure and rewarming rates on oxidative damage immediately and 24-h post-treatment

The rewarming experiment occurred in August and September 2024 (Fig. 1C) and had two main objectives. First, we wanted to measure longitudinal changes in oxidative damage in response to a cold exposure and subsequent recovery. Second, we wanted to determine whether oxidative damage following a cold exposure is primarily caused by the cold temperature itself or the subsequent rate of thermal rewarming, which is difficult, as described below. Measuring oxidative damage immediately after removing lizards from a cold (18°C) incubator presents a methodological challenge: any handling of the lizard inevitably initiates rewarming. Previous work in our lab showed that even the brief time required to remove a lizard from the incubator and measure cloacal temperature (∼10 s) after 24 h at 18°C causes a slight increase in temperature to 18.6±0.4°C. Blood sampling, which takes longer (3.0±1.1 min), would further contribute to rewarming. This inherent rewarming during sampling confounds the ability to isolate the effect of cold exposure alone.

Therefore, to address our second objective and determine the relative contributions of cold exposure versus rewarming to oxidative damage, we designed the experiment to examine how oxidative damage varied with controlled rewarming rates. We hypothesized that if rewarming causes oxidative damage, then faster rewarming rates would lead to higher levels of oxidative damage compared with slower, or near-zero, rates. This approach allowed us to use the rewarming rate slopes to predict oxidative damage at a theoretical rewarming rate of zero (the *y*-intercept). We then compared this predicted zero-rewarming level with oxidative damage levels at the baseline sample. If cold exposure itself is the primary cause of damage, we would predict that oxidative damage at a rewarming rate of zero (when lizard body temperatures remained approximately 18°C) would be significantly higher than at baseline.

For the experimental procedure, on the morning (09:00 h) of the cold temperature exposure, individual lizards were placed in small plastic containers (16.6×12.4×14.1 cm) and put into an incubator set to 18°C in a staggered fashion (beginning at 10:00 h) to allow time for processing the next day (Fig. 1C). Lizards remained at 18°C for 24 h.

To generate a range of rewarming rates (°C min^−1^ g^−1^), which are influenced by factors such as ambient temperature, time spent at that temperature and body condition (larger-bodied lizards warm more slowly than smaller individuals), we employed two different rewarming protocols. Lizards from each population were randomly assigned to one of two protocols, balanced by body mass (Fig. 1C): fast rewarming or slow rewarming. In the fast rewarming protocol, lizards were transferred directly from the 18°C incubator to a 33°C incubator, a temperature that facilitates safe and steady warming (∼1°C min^−1^; Lutterschmidt and Hutchison, 1997). In the slow rewarming protocol, lizards were maintained at 18°C for an additional 10 min before being processed. Thus, only the exposure to the ambient lab temperature and the sampler’s hand affected rewarming.

After 24 h at 18°C, lizards were removed from the incubator and processed according to their assigned rewarming protocol. For both protocols, a blood sample (the rewarming sample) was then collected, followed immediately by a cloacal body temperature measurement using a thermometer (Kizen, JS-24-11). Blood sampling time did not differ significantly between protocols (*t*-test=0.11, *P*=0.92).

Together, these two rewarming protocols, combined with the varying lizard masses (mean±s.d.: 6.9±2.3 g) and the varying time required for blood sampling (3.0±1.1 min) generated substantial variation in rewarming rates (0.10±0.07°C min^−1^ g^−1^) and resulting body temperatures at time of blood sampling (ranging from 19.7 to 31.6°C). This design provided a broad range of rewarming rates to test their effect on oxidative damage for our second objective.

After processing, lizards were returned to their normal terraria with no changes to their food schedule or water availability. Twenty-four hours after the rewarming sample, a third blood sample (post-rewarming sample) was taken to assess longitudinal changes in oxidative damage.

### Blood sampling and processing

Blood samples (∼50 µl) were collected retro-orbitally using a 70 µl heparinized microhematocrit capillary tube. Lizards were restrained in one hand while the blood sample was collected. Lizards were monitored after sampling, and no lizards experienced injuries from blood sampling procedures. Blood samples were briefly stored on ice before being centrifuged for 6 min at 960×***g***. Plasma was removed and 30 µl of NBS:DMSO buffer [90% newborn bovine serum (NBS):10% dimethylsulfoxide] was added to the packed blood cells for resuspension. Cells were stored at −20°C until DNA extraction.

### Oxidative damage assay

In DNA, ROS attack modifies the natural deoxyguanosine nucleoside (dG) to the mutated 8-hydroxy-2′-deoxyguanosine (8-OHdG), which is an established biomarker of DNA oxidative damage (Lunec et al., 2002; Matter et al., 2018). In genomic DNA, 8-OHdG can cause transversion mutations such as G–T or G–A binding. This mutagenic role can influence disease progression and initiates carcinogenesis, making it a biomarker with clinical significance (Bartkova et al., 2006). In studies of natural populations, the accumulation of 8-OHdG can provide insights into how the environment influences aging and survival (Speakman et al., 2015).

To negate any potential effect of the sample handling process on oxidative damage, care was taken to ensure that all samples were treated identically, as detailed in Lichtner et al. (2025). Briefly, to measure oxidative damage, 30 µl of red blood cells were homogenized in DNAzol (ThermoFisher). Protein contaminants were removed and DNA was precipitated from the supernatant using 100% ethanol. The remaining DNA pellet was digested over three 90-min, 37°C incubations. To control for differences in total DNA content among samples, we also measured dG, and the levels of 8-OHdG are expressed as the number of 8-OHdG molecules per 1,000,000 dG (8-OHdG per 10^6^ dG).

Sample preparation and analysis were performed as previously described in Lichtner et al. (2025). Briefly, standard stock solutions of 8-OHdG (1 mg ml^−1^) and internal standard ^15^N_5_-8-OHdG (25 μg ml^−1^) were prepared in water, stored at −20°C and diluted in methanol for working solutions. Samples (50–100 μl) were spiked with 5 μl internal standard, diluted and purified via solid phase extraction on a Phenomenex Strata-X cartridge before drying and reconstituting in 20% methanol.

8-OHdG was quantified using a Sciex QTRAP 6500+ mass spectrometry system coupled with a Sciex EXion HPLC separation system. Separation was performed on a 1.7 μm Acquity UPLC BEH C18 analytical column (2.1×100 mm, Waters, Ireland) with gradient elutions using 0.1% formic acid in molecular grade water (mobile phase A) and acetonitrile (mobile phase B) at 0.3 ml min^−1^. Multiple reaction monitoring was used to detect 8-OHdG (*m*/*z* 284>168) and ^15^N_5_-8-OHdG (*m*/*z* 289>173), with quantification performed using Sciex OS 3.0 software.

dG was analyzed using HPLC-UV as previously described in Lichtner et al. (2025). Briefly, samples were diluted 5- to 10-fold in molecular grade water and 5–10 µl was injected onto a HPLC equipped with a variable wavelength detector. Separation was performed on a Phenomenex C18 Bondclone (10 μm, 3.9×300 mm; Torrance, CA, USA) with an isocratic solvent system of 93% 0.1% formic acid in water and 7% methanol at a flow rate of 1 ml min^−1^ for 20 min. dG was detected at 254 nm, and concentrations were determined using calibration curves based on a dG standard (Lichtner et al., 2025).

### Statistics

We conducted general linear models (GLMs), linear mixed-effects models (LMMs) or generalized linear mixed models (GLMMs) in R (v.4.4.2; https://www.r-project.org/), depending on the structure and distribution of the data. GLMs were used when models did not include random effects. For models including repeated measures on the same individuals, we included ‘individual’ as a random effect to account for the non-independence of data collected on the same individuals. For LMMs, we assessed model assumptions by examining the normality of residuals using histograms and Q–Q plots, and tested for homogeneity of variances using Levene's test. Where these assumptions were violated for the LMM, we opted for a GLMM framework. For LMMs, we used a Gaussian error distribution with an identity link function. For GLMs and GLMMs, we fitted the models using a gamma distribution with a log-link function. All initial full models included the highest-order interactions as described in the relevant sections below. A set of candidate models, encompassing all possible nested combinations of fixed effects derived from the initial full model, was generated. The optimal model was then selected based on the lowest corrected Akaike’s information criterion (AICc) value. AICc is a correction for AIC, specifically designed for situations with small sample sizes relative to the number of parameters. Comprehensive model selection tables, detailing AIC, AICc and Bayesian information criterion values for all candidate models, are provided as Tables S1–S4. Only the final selected model is reported in the results, and for these models *post hoc* pairwise comparisons of estimated marginal means were conducted, with *P*-values adjusted using the Šidák method for multiple comparisons, to evaluate differences among significant main effects and interactions.

In the temperature experiment, we analyzed oxidative damage using a GLMM, with 8-OHdG as the dependent variable, and population, temperature exposure, blood sample (pre-exposure and post-exposure) and all their interactions as fixed effects, and individual as a random effect. Mass was not included as a fixed effect in this analysis, or any subsequent 8-OHdG analysis for two reasons: first, we had no *a priori* predictions for a direct relationship between whole-body mass and 8-OHdG levels; and second, 8-OHdG levels were already normalized to dG content, effectively controlling for cell number and eliminating the need for an additional proxy measure of cell number.

In the rewarming experiment, oxidative damage was analyzed using an LMM, with 8-OHdG as the dependent variable, and population (northern and southern), blood sample (baseline, rewarming, and post-rewarming) and all their interactions as fixed effects, and individual as a random effect. To further explore how mass-specific rewarming rate influenced oxidative damage, a GLM was employed for 8-OHdG levels at the rewarming time point, with population, rewarming rate and their interaction as fixed effects. Finally, to determine whether oxidative damage observed after cold exposure is primarily attributable to the cold temperature itself or the subsequent thermal rewarming, we compared baseline oxidative damage levels with predicted oxidative damage at a rewarming rate of zero (i.e. the predicted 8-OHdG levels if lizards were still at 18°C). Specifically, the 95% confidence intervals of the intercepts from the GLM (representing predicted 8-OHdG at zero warm-up rate for each population) were then compared against the 95% confidence interval of the mean 8-OHdG levels from the baseline samples. Overlap between these confidence intervals was then used to infer the statistical significance of any observed difference.

## RESULTS

### Temperature experiment

**Fig. 2.**
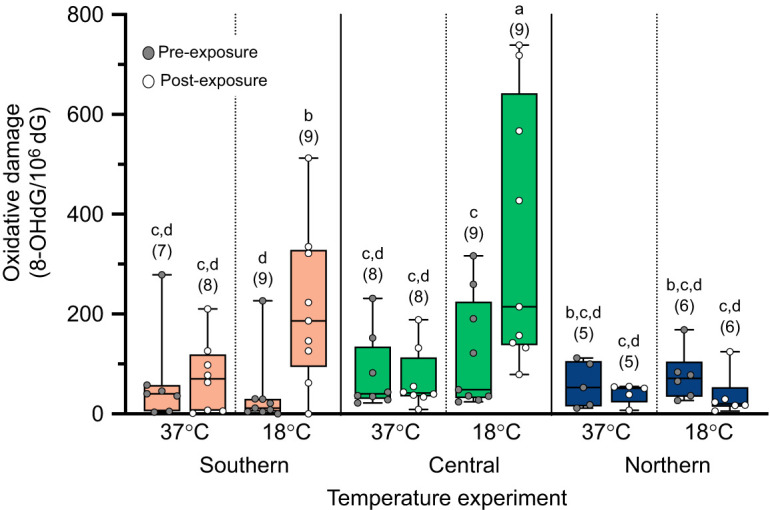
**8-OHdG levels in response to warm and cold thermal exposures in three populations of lizards from the temperature experiment.** Levels of 8-OHdG from pre-exposure (grey circles) and post-exposure (white circles) for the southern (light orange boxes), central (medium green boxes) and northern populations (dark blue boxes). Box and whisker plot show the distribution of the data. Different letters above each box indicate significant differences among groups as determined by the *post hoc* analysis of the significant three-way interaction. In general, each lizard had a pre- and post-exposure sample, but in some cases we did not have enough DNA to measure 8-OHdG values for an individual at one of the two time points (sample sizes shown below significance grouping letters).

In the temperature experiment, a significant three-way interaction was found among temperature exposure, blood sampling time point and population for levels of 8-OHdG, our oxidative damage marker (χ^2^_2_=11.33, *P*=0.003; Fig. 2). Pairwise comparisons revealed no significant differences in 8-OHdG levels between the central and southern populations at any time point or temperature (all *P*>0.05; Fig. 2), thereby justifying their collective grouping as ‘southern populations’ for the discussion. Prior to thermal exposures, 8-OHdG levels did not significantly differ among populations at either temperature (all pairwise, *P*>0.05; Fig. 2). However, the interaction revealed specific patterns of change after exposure. The northern population experienced no significant change in 8-OHdG levels following either the 18°C or 37°C thermal exposures (all pairwise, *P*>0.05; Fig. 2). In contrast, the southern populations exhibited significant increases in 8-OHdG levels after the 18°C exposure (all pairwise, *P*<0.05), but no significant changes in 8-OHdG levels after the 37°C exposure (all pairwise, *P*>0.05; Fig. 2).

### Rewarming experiment

**Fig. 3.**
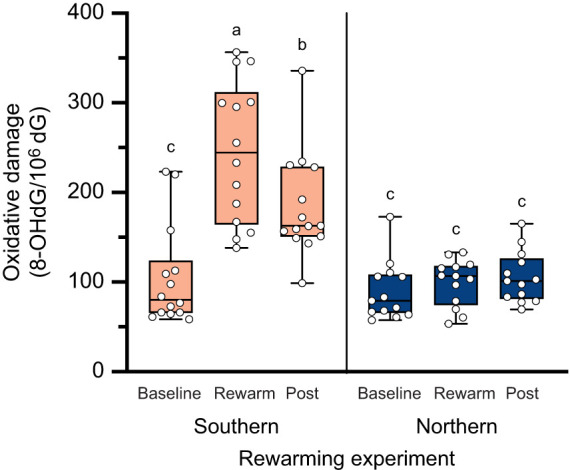
**8-OHdG levels in response to cold thermal exposures, rewarming, and 24-h post-treatment in two populations of lizards from the rewarming experiment.** Levels of 8-OHdG for each individual (white circles) at baseline, rewarming (Rewarm) and post-rewarming (Post) for the southern (light orange boxes) and northern populations (dark blue boxes). Box and whisker plots show the distribution of the data. Different letters above each box denote significant differences among the six sampling groups (baseline southern, rewarm southern, etc.). Groups that share a letter are not significantly different from one another (*N*=14 southern, *N*=13 northern; individuals sampled at all periods).

**Fig. 4.**
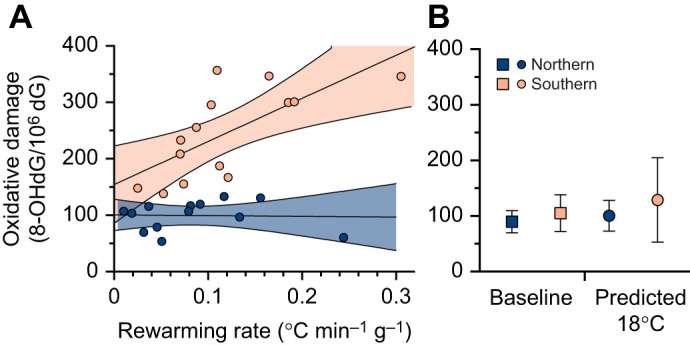
**Effects of rewarming on oxidative damage.** Effects of rewarming on oxidative damage. (A) Relationships between rewarming rate (°C min^−1^ g^−1^) and 8-OHdG levels for individuals from northern (dark blue circles) and southern (light orange circles) populations at the rewarming sample. The shaded regions indicate the 95% confidence intervals of the regression lines. (B) Comparison of mean 8-OHdG levels at baseline (circles) versus predicted levels at a warm-up rate of 0 (i.e. predicted 18°C, squares) for southern (light orange) and northern (dark blue) populations. Error bars represent 95% confidence intervals of the means.

In the rewarming experiment, for 8-OHdG levels, the top model revealed a significant two-way interaction between population and blood sampling time point (*F*_2,50.1_=14.4, *P*<0.001; Fig. 3). Similar to the temperature experiment, the southern population had an increase in 8-OHdG after the 18°C exposure (all pairwise, *P*<0.05; Fig. 3), whereas 8-OHdG levels in the northern population did not differ between blood sampling time points (all pairwise, *P*>0.5; Fig. 3). In the southern population, the increased levels following the 18°C exposure significantly decreased after 24 h (Tukey’s HSD, *P*<0.05; Fig. 3). Interestingly, when exploring the effects of rewarming rate, the top model identified a significant two-way interaction between population and mass-specific rewarming rate (χ^2^_1_=6.0, *P*=0.01), with faster rewarming rates associated with higher levels of 8-OHdG in individuals from the southern population, but no effect of the rate of rewarming on 8-OHdG levels in the northern population (Fig. 4A). In addition, 8-OHdG at a warm-up rate of zero (i.e. the predicted 8-OHdG levels at 18°C) was not significantly different from baseline 8-OHdG levels in either the northern or southern populations, as indicated by the overlap of their respective 95% confidence intervals (Fig. 4B).

## DISCUSSION

Our study examined oxidative damage responses of a widely distributed lizard species to day-long temperature exposures. Following a warm exposure, none of the populations accumulated oxidative damage. However, following a cold exposure, the southern population accrued oxidative damage, while the northern population did not. This trend remained consistent across both the temperature and rewarming experiments, indicating robust population patterns despite slightly different experimental conditions, individuals and study years. Additionally, we examined whether the accumulation of oxidative damage in the southern population was a result of the cold temperature itself or the subsequent rate of rewarming, and found evidence for the latter. Finally, the cold-induced oxidative damage in the southern population appears to be transient, as levels dropped 24 h after the cold exposure.

Our study supports the climate variability hypothesis, providing two new lines of evidence that broaden its scope. First, although traditional studies have explored the hypothesis across species, to our knowledge, ours is the first to provide intraspecific support within a vertebrate species. Second, although some recent work has examined the hypothesis by exploring mitochondrial performance across different temperatures (Havird et al., 2020), the present study is the first to examine temperature-induced oxidative damage.

### Oxidative stress in ectotherms: the role of temperature

Ectotherm body temperatures naturally fluctuate with ambient temperatures throughout the day, with temperature variation becoming more pronounced in temperate regions (Kefford et al., 2022). The connection between body temperature and oxidative damage in vertebrate ectotherms is of substantial interest, particularly within the framework of the climate variability hypothesis, which suggests that adaptation to variable thermal environments can shape an organism's physiological responses to temperature fluctuations. General patterns of how ectotherm body temperature relates to oxidative damage are based on the combined influence of two factors (as reviewed by Ritchie and Friesen, 2022). First, oxidative stress generally increases as temperature deviates further from an organism's optimal temperature, whether higher or lower (Ritchie and Friesen, 2022). However, the duration of temperature exposure modifies this pattern, with cold temperatures more likely to induce oxidative stress after long-term exposure (Bury et al., 2018; Fitzpatrick et al., 2019; Loughland and Seebacher, 2020) and warmer temperatures more likely to elicit oxidative damage after acute exposure (Loughland and Seebacher, 2020; Madeira et al., 2016). Notably, these patterns do not always hold, and responses vary both within and among taxa (Birnie-Gauvin et al., 2017), with some fish (Hemmer-Brepson et al., 2013), amphibians (Liu et al., 2018) and reptiles (Zhang et al., 2017) showing no oxidative stress response to temperature shifts.

To our knowledge, three studies to date have explored cold temperature exposure and oxidative damage in vertebrate ectotherms (Bury et al., 2018; Fitzpatrick et al., 2019; Zhang et al., 2017), and most of them found that oxidative stress increased with cold temperature exposure (but see Zhang et al., 2017). For example, Bury et al. (2018) reported that grass snakes (*Natrix natrix*) maintained at 18°C for 6 months had higher levels of hydroperoxides, an indicator of oxidative damage, compared with snakes maintained at 32°C. Our study in prairie lizards also showed an increase in oxidative damage, albeit after a much shorter acclimation period. However, our rewarming experiment revealed that the accumulation of oxidative damage appears to result not from the cold temperature itself, but from the subsequent rewarming. Indeed, individuals with rewarming rates closest to zero had levels of oxidative damage that were not significantly different from baseline levels (Fig. 4B). To our knowledge, the previously mentioned studies that found increased oxidative stress after cold exposure did not take into consideration the possible effects of rewarming during sampling. This suggests that the increased oxidative stress found in these studies is most likely due to rewarming to normal ambient temperatures.

This helps to place our results into the larger body of literature on temperature and oxidative stress across vertebrate ectotherms, and potentially suggests a more nuanced relationship between cold exposure, rewarming and oxidative damage. Similar to our results, a number of studies, mostly in fish, report that acute rewarming causes oxidative stress (Bagnyukova et al., 2007; Madeira et al., 2013, 2016; Vinagre et al., 2014). Further supporting the importance of acute rewarming in generating oxidative stress is a study in Chinese soft-shelled turtles (*Pelodiscus sinensis*) that did not observe an increase in oxidative stress after cold temperatures (Zhang et al., 2017). In that study, turtles were exposed to 8°C for 12 h, and then rewarmed to 28°C for 24 h to assess oxidative stress responses. Although the cold exposure did not affect oxidative stress, rewarming did increase oxidative stress (Zhang et al., 2017). In addition, a study exploring warming after winter hibernation in marsh frogs (*Rana ridibunda*) reported that a rapid rewarming of 1 h increased oxidative damage in multiple tissues (Bagnyukova et al., 2003). Our study showed that the increase in oxidative stress could occur in a drastically shorter time frame, within minutes. Taken together, our results and those from the literature suggest that it is possible that some of the oxidative stress attributed to cold exposure in vertebrate ectotherms may, in fact, result from the acute rewarming that follows, rather than the cold temperatures themselves.

### Possible mechanisms underlying increased oxidative damage during rewarming in southern prairie lizard populations

As mentioned previously, the electron transfer system within the mitochondria serves as the main source of ROS production. The rate of ROS generation is closely tied to the proton motive force, the proton gradient established by the electron transfer system, with a high proton motive force increasing the likelihood of electron leak. These escaped electrons may prematurely reduce molecular oxygen, leading to ROS formation.

Why, then, should ROS be produced during acute temperature increases in ectotherms? One major contributing factor is that rising temperatures elevate metabolic rates, leading to increased oxygen consumption and, consequently, greater ROS production (Halliwell and Gutteridge, 2015; Schulte, 2015). Additionally, abrupt temperature shifts in ectotherms, such as a rapid shift from cold to warm conditions, can lead to sudden changes in circulation. In cold temperatures, blood flow and oxygen delivery are restricted, but upon warming, circulation is rapidly restored (Crawshaw, 1979). This sudden increase in oxygen availability can, in turn, drive metabolic rates higher and lead to a sharp rise in proton motive force. Such a process resembles well-documented reperfusion events, where tissues experience a surge of oxygen after a period of deprivation, triggering excessive ROS production (Cadenas, 2018).

Recall that ROS have detrimental effects by causing deleterious damage, but also beneficial effects, serving as important signaling molecules (Lichtner et al., 2025). One possibility is that the accumulation of damage during rewarming in the southern population acts as a cellular signal (Picard and Shirihai, 2022), indicating changes occurring within the mitochondrial electron transfer system. However, even if this was the case, the level of oxidative damage accumulation within these populations was high. From baseline to rewarming, there was a 5-fold median increase in oxidative damage in the southern population. Providing direct ecological context for this increase is challenging, given the limited cellular 8-OHdG research in ecology generally and the absence of such studies in reptiles specifically. Nevertheless, the human biomedical literature offers some comparative insight; for instance, cancerous tissue, with its altered metabolism and high ROS production, often exhibits 8-OHdG levels that are 1.5-fold higher than normal tissue (Okamoto et al., 1994; Xu et al., 2013). In addition, other studies of temperature-induced oxidative damage also show increases in lipid oxidation. Chinese pond turtles (*Mauremys reevesii*) and Asian yellow pond turtles (*Mauremys mutica*) experiencing heat waves have a 1.5-fold increase in lipid oxidative damage (Li et al., 2021; Tao et al., 2022), and gilt-head bream (*Sparus aurata*) rewarming at a rate of 1°C h^−1^ showed a 3.2-fold increase in lipid damage (Madeira et al., 2016). Such high levels of damage raise questions about whether the oxidative stress experienced by the southern population is within a tolerable range or whether it instead exceeds a threshold that could impair mitochondrial function and overall physiological resilience.

To gain further insight into possible long-term effects, we measured 8-OHdG levels longitudinally in the rewarming experiment, tracking oxidative damage from a baseline sample taken 1 month before temperature exposures, immediately after rewarming and again 24 h later. We observed a significant decrease in 8-OHdG levels at the post-rewarming time point in the southern population, though they had not yet recovered to baseline values. This suggests that although this population does not initially resist oxidative damage, it may have the capacity to recover through base-excision repair mechanisms. Even if the damage is transient, resources devoted to repairing or replacing damaged molecules come at the expense of other essential cellular processes, so the energetic burden could be significant. For example, Salin et al. (2015) found that increasing mitochondrial efficiency (reducing ROS production) in brown trout (*Salmo trutta*) came at the cost of growth. Future work should focus on quantifying these trade-offs to investigate how the energetic burden of oxidative damage repair influences broader physiological performance.

Additionally, it is important to recognize that our study design cannot distinguish between the repair of damaged DNA and the production of undamaged cells by cell turnover. Following blood collection after the rewarming sample, lizards may have responded to decreased oxygen delivery by producing new red blood cells, which could have artificially lowered the observed levels of 8-OHdG. If this is the case, the true extent of oxidative damage may be masked by new cells rather than repair. Taken together, these findings suggest that even if the southern population responds to thermal fluctuations through important ROS signaling, the initial accumulation of oxidative damage may still outweigh any potential benefits, ultimately imposing physiological costs. The longitudinal sampling approach used in this study provides valuable insights into oxidative stress dynamics, highlighting the importance of incorporating similar designs in future research. Additionally, future studies could investigate whether this oxidative damage has functional consequences by assessing mitochondrial function using high-resolution respirometry.

### Why might northern prairie lizard populations escape temperature-induced oxidative damage?

In our experiments, we used a common garden experimental approach to minimize environmental effects, which is a standard method for investigating the genetic basis of trait differences among populations (De Villemereuil et al., 2016). Although this approach strongly suggests a genetic component to the observed differences, we cannot rule out the influence of developmental plasticity, which could alter gene expression. Before exploring potential mechanistic explanations for why the northern populations may be able to limit temperature-induced oxidative damage, it is important to clarify that oxidative damage is not the same as ROS levels. Instead, oxidative damage represents the downstream byproduct of ROS attacking biomolecules such as DNA. Therefore, our finding that the northern population exhibited resistance to oxidative damage accumulation following a cold exposure and rewarming can have multiple, non-mutually exclusive mechanistic explanations. In general, these mechanisms can be divided into two groups: those that occur after ROS generation, and those that occur before ROS generation, through the prevention of their formation in the first place.

Mechanisms that act on ROS after they have already been generated include both neutralizing ROS before they can cause damage, and removing or repairing already damaged molecules (Monaghan et al., 2009). Both of these mechanisms rely, in part, on enzymes. Because most enzymes are temperature-dependent, if the northern population is better adapted to temperature fluctuations as the climate variability hypothesis would predict, one possibility is that their antioxidant, repair and removal enzymes may have expanded functional thermal ranges. This would favor enzymatic function at lower temperatures in preparation for subsequent warming stressors, allowing for better ROS scavenging or damage repair. This idea aligns closely with the established ‘preparation for oxidative stress’ hypothesis, which suggests that during periods of reduced metabolic function, such as cold temperatures in ectotherms, organisms can increase antioxidant capacity in anticipation of oxidative stress upon returning to basal metabolic rates (Moreira et al., 2023; Giraud-Billoud et al., 2019, 2024).

In contrast, a potentially more efficient upstream mechanism involves reducing oxidative damage by limiting ROS production from the start. One possible mechanism to prevent ROS production is to increase mitochondrial uncoupling through insertion of uncoupling proteins into the mitochondrial membrane. To date, studies exploring uncoupling proteins in vertebrate ectotherms are limited, though uncoupling protein expression after cold exposure has been found in fish (Jastroch et al., 2007; Tseng et al., 2011), amphibians (Issartel et al., 2009) and reptiles (Rey et al., 2008; Schwartz et al., 2008). A second possible mechanism through which ROS levels could be altered is through the modification of the functional proteins of the electron transfer system itself. Mitochondrial genes have been found to be more sensitive to temperature shifts compared with nuclear genes, which could influence mitochondrial performance and ROS production. For example, He et al. (2024) found changes in mitochondrial gene expression in cold-tolerant frogs that could lead to increased uncoupling and lower ROS production. Even more relevant to the possible thermal adaptations between our lizard populations are the findings of Jin et al. (2022), who studied two subspecies of tiger frogs (*Hoplobatrachus rugulosus*), one of which is adapted to colder environments and can survive temperatures that are lethal to the other subspecies. After cold exposure, the cold-tolerant subspecies decreased mitochondrial gene expression that altered electron transfer system proteins in a way that reduced the proton motive force. Although oxidative stress was not measured in that study, the authors suggest that these changes in gene expression could affect ROS production (Jin et al., 2022).

Taken together with our findings, this suggests that broadly distributed ectotherms species may evolve population-specific mitochondrial responses to the thermal range they experience most often, contributing to variation in oxidative stress resistance across different climatic regions. More broadly, temperature may exert strong selective pressure on mitochondrial function, leading to population divergence over time. This aligns with Sokolova (2023), who proposed that mitochondrial function and organismal thermal tolerance evolve under similar selective pressures, reinforcing the idea that temperature-driven mitochondrial adaptations could contribute to speciation events in widely distributed ectothermic species.

### Conclusions

Our study highlights how temperature, a major challenge for ectotherms, has shaped adaptive responses in populations facing distinct thermal environments. Previous common garden studies on these lizards have revealed adaptive phenotypes to colder climates, including increased body condition (Robbins and Hegdahl, 2024), faster growth rates (Robbins and Hegdahl, 2024) and accelerated embryonic development (Haussmann, 2024; Lenard and Gifford, 2019). Our findings suggest that increased resistance to oxidative damage may represent another such adaptation, contributing to improved cold tolerance. In particular, the northern population accumulated less oxidative damage after cold exposure and rewarming. Our findings offer support for the climate variability hypothesis, which predicts that broader physiological tolerances evolve in species from more thermally variable environments, and suggests that this framework can be extended to populations within species. Further exploration of gene expression changes and mitochondrial efficiency under thermal stress could reveal the molecular mechanisms underlying these patterns.

Populations exposed to greater thermal variability may face stronger selection on mitochondrial function and oxidative stress pathways. Over time, these divergent thermal regimes could drive evolutionary changes in metabolism and potentially contribute to speciation in broadly distributed ectotherms. We caution that overlooking intraspecific variation in thermal tolerance can lead to misleading predictions about species' responses to environmental change, and others have emphasized the need for longitudinal studies, like ours, that explicitly examine variation in physiological resilience among populations (Bennett et al., 2019). Understanding how organisms may have evolved and adapted to local climates can provide insight for predicting evolutionary trajectories in response to rapid thermal shifts in an increasingly variable and warming world.

## Supplementary Material

10.1242/jexbio.251040_sup1 Supplementary information
