# Supplementary material for: Intraspecific support for the climate variability hypothesis: oxidative damage in lizards after acute temperature exposure
Source: J Exp Biol. 2025 Sep 26;228(18):jeb251040. doi: 10.1242/jeb.251040 (PMC12517349; doi:10.1242/jeb.251040)
Supplement: Supplementary information [file jexbio-228-251040-s1.pdf]

These tables present the results of the model selection process for identifying the best-fitting linear models. Models were ranked using the corrected Akaike Information Criterion (AICc), with the model exhibiting the lowest AICc value considered the most parsimonious and best-supported. In each case, model selection was performed using the dredge function from the MuMIn package in R, on the full model.

**Table S1**. Summary of the Generalized Linear Mixed Models (GLMMs) evaluating metabolic rate (MR) as a function of Population (Pop), Temperature Temp), and their interaction, and Mass. Individual identity was treated as a random effect, with a Gamma distribution and log link. The + symbol in the variable columns indicates that the corresponding term was included in that specific model. Models are ranked using AICc, wirth the best model at the top.

| Intercept | Mass | Pop | Temp | Pop:Temp | AIC | BIC | df | logLik | AICc | delta | weight |
|---|---|---|---|---|---|---|---|---|---|---|---|
| **0.13070** | **-0.16450** | | **+** | | **-59.01** | **-46.80** | **5** | **34.506** | **-58.3** | **0.00** | **0.660** |
| 0.41350 | -0.16630 | + | + | | -57.93 | -40.83 | 7 | 35.966 | -56.5 | 1.78 | 0.271 |
| 0.46760 | -0.17080 | + | + | + | -55.88 | -33.90 | 9 | 36.942 | -53.5 | 4.77 | 0.061 |
| -1.30800 | | | + | | -49.45 | -39.68 | 4 | 28.726 | -49.0 | 9.30 | 0.006 |
| -1.17600 | | + | + | | -47.25 | -32.59 | 6 | 29.625 | -46.2 | 12.08 | 0.002 |
| -1.15200 | | + | + | + | -44.25 | -24.71 | 8 | 30.126 | -42.4 | 15.90 | 0.000 |
| -0.82980 | 0.09389 | | | | 183.00 | 192.80 | 4 | -87.512 | 183.5 | 241.78 | 0.000 |
| 0.05303 | | | | | 186.20 | 193.50 | 3 | -90.090 | 186.5 | 244.73 | 0.000 |
| -1.10300 | 0.11870 | + | | | 186.20 | 200.80 | 6 | -87.088 | 187.3 | 245.51 | 0.000 |
| 0.11590 | | + | | | 189.80 | 202.00 | 5 | -89.905 | 190.6 | 248.82 | 0.000 |

Journal of Experimental Biology • Supplementary information

**Table S2.** Summary of the Generalized Linear Mixed Models (GLMMs) evaluating oxidative damage (OxD) as a function of Population (Pop), Temperature (Temp), Blood sample (Sam), and their interactions. Individual identity was treated as a random effect, with a Gamma distribution and log link. The + symbol in the variable columns indicates that the corresponding fixed effect term(s) were included in that specific model. Models are ranked using AICc, with the best model at the top.

| Intercept | Pop | Sam | Temp | Pop: Sam | Pop: Temp | Sam: Temp | Pop:Sam: Temp | AIC | BIC | df | logLik | AICc | delta | weight |
|---|---|---|---|---|---|---|---|---|---|---|---|---|---|---|
| **4.591** | **+** | **+** | **+** | **+** | **+** | **+** | **+** | **964.3** | **998.8** | **14** | **-468.156** | **970.1** | **0.00** | **0.731** |
| 4.651 | + | + | + | + | + | + | | 969.0 | 998.6 | 12 | -472.512 | 973.2 | 3.09 | 0.156 |
| 4.415 | + | + | + | + | | + | | 972.0 | 996.6 | 10 | -475.983 | 974.9 | 4.71 | 0.069 |
| 4.197 | | + | + | | | + | | 977.0 | 991.8 | 6 | -482.512 | 978.1 | 7.93 | 0.014 |
| 4.932 | + | + | + | + | + | | | 974.9 | 1002.0 | 11 | -476.465 | 978.4 | 8.30 | 0.012 |
| 4.636 | + | + | + | + | | | | 976.9 | 999.1 | 9 | -479.460 | 979.3 | 9.11 | 0.008 |
| 4.506 | + | + | + | | | + | | 978.0 | 997.8 | 8 | -481.015 | 979.9 | 9.73 | 0.006 |
| 4.690 | + | + | + | | + | + | | 978.8 | 1003.0 | 10 | -479.407 | 981.7 | 11.56 | 0.002 |
| 4.415 | + | + | | + | | | | 981.1 | 1001.0 | 8 | -482.567 | 983.0 | 12.83 | 0.001 |
| 4.484 | | + | + | | | | | 983.0 | 995.3 | 5 | -486.478 | 983.7 | 13.55 | 0.001 |
| 4.812 | + | + | + | | | | | 983.3 | 1001.0 | 7 | -484.639 | 984.7 | 14.55 | 0.001 |
| 5.022 | + | + | + | | + | | | 983.6 | 1006.0 | 9 | -482.781 | 985.9 | 15.75 | 0.000 |
| 5.329 | + | | + | | + | | | 985.5 | 1005.0 | 8 | -484.765 | 987.4 | 17.23 | 0.000 |
| 5.152 | + | | + | | | | | 986.5 | 1001.0 | 6 | -487.227 | 987.5 | 17.36 | 0.000 |
| 4.834 | | | + | | | | | 987.4 | 997.2 | 4 | -489.692 | 987.9 | 17.73 | 0.000 |

Journal of Experimental Biology • Supplementary information

| | | | | | | | | | | | | | | |
|---|---|---|---|---|---|---|---|---|---|---|---|---|---|---|
| 4.556 | + | + | | | | | | 990.7 | 1005.0 | 6 | -489.326 | 991.7 | 21.56 | 0.000 |
| 4.139 | | + | | | | | | 991.4 | 1001.0 | 4 | -491.699 | 991.9 | 21.74 | 0.000 |
| 4.984 | + | | | | | | | 996.1 | 1008.0 | 5 | -493.034 | 996.8 | 26.66 | 0.000 |
| 4.528 | | | | | | | | 999.5 | 1007.0 | 3 | -496.725 | 999.7 | 29.59 | 0.000 |

Journal of Experimental Biology • Supplementary information

**Table S3.** Summary of the Linear Mixed Models (LMMs) evaluating Oxidative Damage (OxD) as a function of Population (Pop), Blood Sample (Sam), and their interaction. Models were ranked using the corrected Akaike Information Criterion (AICc), with the model exhibiting the lowest AICc value considered the most parsimonious and best-supported.

| Intercept | Pop | Sam | Pop:Sam | AIC | BIC | df | logLik | AICc | delta | weight |
|---|---|---|---|---|---|---|---|---|---|---|
| **89.75** | **+** | **+** | **+** | **873.9** | **893.1** | **8** | **-428.965** | **875.9** | **0.00** | **1** |
| 56.22 | + | + | | 894.0 | 908.4 | 6 | -441.017 | 895.2 | 19.24 | 0 |
| 98.22 | + | | | 913.0 | 922.6 | 4 | -452.487 | 913.5 | 37.57 | 0 |
| 100.50 | | + | | 917.2 | 929.2 | 5 | -453.610 | 918.0 | 42.09 | 0 |
| 141.00 | | | | 934.4 | 941.6 | 3 | -464.219 | 934.7 | 58.82 | 0 |

**Table S4.** Summary of the Generalized Linear Models (GLMs) evaluating Oxidative Damage (OxD) as a function of Population (Pop), Rewarming Rate (Rate), and their interaction. Models were ranked using the corrected Akaike Information Criterion (AICc), with the model exhibiting the lowest AICc value considered the most parsimonious and best-supported.

| Intercept | Pop | Rate | Pop:Rate | AIC | BIC | df | logLik | AICc | delta | weight |
|---|---|---|---|---|---|---|---|---|---|---|
| 4.988 | + | 4.345 | + | 283.1 | 289.6 | 5 | -136.564 | 286.0 | 0.00 | 0.735 |
| 5.255 | + | 2.038 | | 287.1 | 292.2 | 4 | -139.525 | 288.9 | 2.88 | 0.174 |
| 5.504 | + | | | 289.1 | 293.0 | 3 | -141.553 | 290.1 | 4.16 | 0.092 |
| 4.680 | | 4.570 | | 313.8 | 317.7 | 3 | -153.919 | 314.9 | 28.90 | 0.000 |
| 5.166 | | | | 319.3 | 321.8 | 2 | -157.626 | 319.8 | 33.77 | 0.000 |

Journal of Experimental Biology • Supplementary information
